# Diffusion histogram profiles predict molecular features of grade 4 in histologically lower-grade adult diffuse gliomas following WHO classification 2021

**DOI:** 10.1007/s00330-023-10071-x

**Published:** 2023-08-15

**Authors:** Ryo Kurokawa, Akifumi Hagiwara, Mariko Kurokawa, Benjamin M. Ellingson, Akira Baba, Toshio Moritani

**Affiliations:** 1 Division of Neuroradiology, Department of Radiology, University of Michigan, 1500 E. Medical Center Dr, Ann Arbor, MI 48109 USA; 2 Department of Radiology, Graduate School of Medicine, The University of Tokyo, 7-3-1 Hongo, Bunkyo-ku, Tokyo, 113-8655 Japan; 3 Department of Radiology, Juntendo University School of Medicine, 2-1-1 Hongo, Bunkyo-ku, Tokyo, 113-8421 Japan; 4 UCLA Brain Tumor Imaging Laboratory, Department of Radiological Sciences, David Geffen School of Medicine, University of California Los Angeles, 924 Westwood Blvd., Suite 615, Los Angeles, CA 90024 USA

**Keywords:** Glioma, World Health Organization, Diffusion magnetic resonance imaging, Entropy, Magnetic resonance imaging

## Abstract

**Objectives:**

In the latest World Health Organization classification 2021, grade 4 adult diffuse gliomas can be diagnosed with several molecular features even without histological evidence of necrosis or microvascular proliferation. We aimed to explore whole tumor histogram-derived apparent diffusion coefficient (ADC) histogram profiles for differentiating between the presence (Mol-4) and absence (Mol-2/3) of grade 4 molecular features in histologically lower-grade gliomas.

**Methods:**

Between June 2019 and October 2022, 184 adult patients with diffuse gliomas underwent MRI. After excluding 121 patients, 18 (median age, 64.5 [range, 37–84 years]) Mol-4 and 45 (median 40 [range, 18–73] years) Mol-2/3 patients with histologically lower-grade gliomas were enrolled. Whole tumor volume-of-interest-derived ADC histogram profiles were calculated and compared between the two groups. Stepwise logistic regression analysis with Akaike’s information criterion using the ADC histogram profiles with *p* values < 0.01 and age at diagnosis was used to identify independent variables for predicting the Mol-4 group.

**Results:**

The 90th percentile (*p *< 0.001), median (*p *< 0.001), mean (*p *< 0.001), 10th percentile (*p *= 0.014), and entropy (*p *< 0.001) of normalized ADC were lower, and kurtosis (*p *< 0.001) and skewness (*p *= 0.046) were higher in the Mol-4 group than in the Mol-2/3 group. Multivariate logistic regression analysis revealed that the entropy of normalized ADC and age at diagnosis were independent predictive parameters for the Mol-4 group with an area under the curve of 0.92.

**Conclusion:**

ADC histogram profiles may be promising preoperative imaging biomarkers to predict molecular grade 4 among histologically lower-grade adult diffuse gliomas.

**Clinical relevance statement:**

This study highlighted the diagnostic usefulness of ADC histogram profiles to differentiate histologically lower grade adult diffuse gliomas with the presence of molecular grade 4 features and those without.

**Key Points:**

*• ADC histogram profiles to predict molecular CNS WHO grade 4 status among histologically lower-grade adult diffuse gliomas were evaluated.*

*• Entropy of ADC and age were independent predictive parameters for molecular grade 4 status.*

*• ADC histogram analysis is useful for predicting molecular grade 4 among histologically lower-grade gliomas.*

**Supplementary Information:**

The online version contains supplementary material available at 10.1007/s00330-023-10071-x.

## Introduction

Gliomas are the most frequent primary brain tumors in adults with an overall age-adjusted population incidence rate of 4.67–5.73 per 100,000, without remarkable change over the past two decades [1, 2]. Adult diffuse gliomas are graded as CNS WHO grades 2, 3, or 4 according to the degree of malignancy; the higher the grade, the more malignant, and the poorer the prognosis. The primary treatment for IDH-mutant grade 2 and 3 diffuse gliomas is maximal safe resection followed by active surveillance, or radiation followed by procarbazine, lomustine, and vincristine, while radiation with adjuvant temozolomide is often used in the treatment of IDH-mutant astrocytoma, grade 4 and IDH-wildtype glioblastomas (grade 4) [3]. Despite the development of therapeutics, the prognosis for grade 4 gliomas remains unfavorable. The reported median survival of grades 2, 3, and 4 gliomas are approximately 5–17 years, 2–8 years, and <1 year, respectively [1].

Recent advances in molecular genetics have allowed for a more concise definition of CNS tumors based on molecular genetic features. The diagnostic criteria for adult diffuse gliomas now rely on the presence or absence of an isocitrate dehydrogenase (IDH) gene mutation and 1p/19q codeletion, which were adopted in the 2016 edition of WHO classification [4], with the addition of several updates in the latest WHO classification in 2021 (WHO CNS5) [5]. For example, a diagnosis of IDH-wildtype grade 4 diffuse glioma (glioblastoma) includes 3 new molecular genetic criteria: concurrent gain of the whole chromosome 7 and loss of the whole chromosome 10 (+7/−10), telomerase reverse transcriptase (TERT) promoter mutation, and epidermal growth factor receptor (EGFR) amplification. Meanwhile, oligodendrogliomas are classified only into grade 2 or 3 [6]. Due to its rarity, an IDH-wildtype lower-grade (grade 2 or 3) astrocytoma is no longer regarded as a distinct tumor type in WHO CNS5 [6].

Due to these recent revisions and reclassifications of WHO criteria, a diagnosis of grade 4 adult diffuse gliomas should be made even if there is no histological evidence of necrosis or microvascular proliferation. The presence of grade 4 diffuse glioma among tumors diagnosed as lower-grade glioma in previous criteria evokes a challenging problem in radiological diagnosis. Traditionally, a rim-enhancing mass with central necrosis has been considered a typical appearance for grade 4 diffuse glioma, including IDH-wildtype glioblastomas and grade 4 IDH-mutant astrocytomas [7, 8]. However, even tumors without imaging evidence of necrosis could be diagnosed as grade 4 diffuse gliomas according to WHO CNS5 [5].

Despite the difficulty of imaging diagnosis, it is meaningful to diagnose these “molecular” grade 4 diffuse gliomas because these updates are based on advances in molecular genetic findings that revealed histologically lower-grade gliomas with these molecular abnormalities have a prognosis similar to those of histological grade 4 diffuse gliomas [9–11]. Moreover, these molecular genetic features are potential targets for molecularly targeted therapies currently under development, although the results have not yet been satisfactory [12, 13]. Therefore, imaging biomarkers on preoperative MRI for predicting molecular grade 4 diffuse glioma have been warranted to formulate a treatment strategy. Significant efforts have been made to differentiate grade 4 and lower-grade diffuse gliomas, and DWI has provided useful imaging biomarkers including ADC [14–17]. However, imaging biomarkers that can be applied for glioma grading in accordance with WHO CNS5 have been largely unknown except for recently reported gyriform infiltration on fluid-attenuated inversion recovery (FLAIR) imaging [18]. The gyriform infiltration was defined as an elective cortical hyperintensity on FLAIR images without involvement of the underlying white matter and without contrast enhancement [18]. This sign was reportedly suggestive of molecular grade 4 diffuse glioma compared with its lower grade counterparts, but its diagnostic performance has not been fully validated because it was reported only from one single institution [18].

The aim of this study was to explore ADC histogram profiles that are useful for predicting molecular CNS WHO grade 4 status among histologically lower-grade adult diffuse gliomas. We also evaluated the diagnostic performance of gyriform infiltration.

## Materials and methods

The institutional review board approved this single institutional retrospective study, and consent exemption was obtained. Data were acquired in compliance with all the applicable Health Insurance Portability and Accountability Act regulations. Data were de-identified before any analysis.

### Patients

The electronic database of our hospital was searched, yielding 184 patients with pathologically proven adult-type diffuse glioma who fulfilled the following inclusion criteria, between June 2019 and October 2022.

The inclusion criteria were as follows: adult patients ≥18 years old; known molecular and histological findings necessary for diagnosis in accordance with WHO CNS5; and MRI, including FLAIR, pre-and post-contrast enhanced T1-weighted imaging, and DWI with b-values of 0 and 1000 s/mm^2^, was performed in our hospital within 3 months before biopsy/surgery.

The exclusion criteria were as follows: tumors with histological evidence of CNS grade 4 (*n* = 82); recurrent/residual tumors (*n* = 38); and MRI was performed after treatment initiation (*n* = 1).

Finally, 63 patients (median age 45 [range, 18–84] years; 31 men) were enrolled in the study.

### MRI scanning protocol

Brain MRI examinations were performed using 1.5-T (*n* = 13) and 3-T (*n* = 50) MRI systems (Ingenia (1.5 T, 3 T), Achieva (1.5 T): Philips Healthcare; MAGNETOM Sola (1.5 T), Skyra (3 T), MAGNETOM Vida (3 T): Siemens) in the supine position. MRI acquisition protocols are summarized in Table 1.

**Table 1:** MRI acquisition protocol

	FLAIR	Pre-and post-contrast fat-sat T1WI	DWI (b = 0, 1000 s/mm^2^)
Plane	Axial	Axial	Axial
Repetition time (ms)	8500–11,000	500–2300	3500–5900
Echo time (ms)	105–140	5–20	58.2–91.2
Flip angle (degree)	90–150	69–125	90–180
Number of excitations	1–2	1–2	1–2
Slice thickness/increment (mm)	4–5/4.4–6	4–5/4.4–6	4–5/4.4–5
Field of view (mm)	228–252	160–240	227–252
Matrix	320 × 310–560 × 560	188 × 188–320 × 320	176 × 176–320 × 320

### Data analysis

The following demographic and clinical data of the patients were collected: age at diagnosis, sex, and period between MRI and diagnosis.

### MR imaging evaluation on FLAIR images

The presence or absence of a gyriform infiltration was evaluated on FLAIR images by two experienced board-certified radiologists by consensus. The radiologists were blinded to the molecular group of the patients when they interpreted the MR images.

### Postprocessing of MRI data

ADC maps were calculated from the DWI data. Pre-contrast T1-weighted images, FLAIR images, and ADC maps were registered to post-contrast T1-weighted images for subsequent analyses (tkregister2; Freesurfer; Massachusetts General Hospital, Harvard Medical School; https://surfernmr.mgh.harvard.edu.). Three spherical volumes of interest (VOIs), each 5 mm in diameter, were put in the central semiovale in the anterior, middle, and posterior area approximately 3 mm above the upper end of the lateral ventricles contralateral to the tumor by an experienced board-certified radiologist using ITK-SNAP software (version 3.8.0; http://www.itksnap.org) [19, 20]. The mean value of the mean ADC values within these 3 VOIs was used to normalize the ADC map. Signal intensity of the post- and pre-contrast T1-weighted images was normalized, and T1-weighted digital subtraction maps were created for tumor segmentation using the previously reported method [21].

### Volume-of-interest analysis

Three mutually exclusive VOIs were defined using a semi-automated thresholding method using the previously reported method [21, 22]. They included the following: (a) contrast-enhancing tumor; (b) cystic components defined by T1-weighted subtraction maps; and (c) hyperintense regions on FLAIR images (non-enhancing tumor) excluding areas of contrast enhancement and cystic components. Contrast-enhancing and non-enhancing tumor VOIs were combined to calculate the histogram profiles of ADC values within the tumor, providing the following set of parameters: maximum, 90th percentile, median, mean, 10th percentile, minimum, kurtosis, skewness, and entropy. The histogram parameters were calculated by Matlab (release 2018a, MathWorks).

### Statistical analysis

Age (years), the period between pretreatment MRI and biopsy/surgery (day), and ADC histogram profiles (maximum, 90th percentile, median, mean, 10th percentile, minimum, kurtosis, skewness, and entropy of normalized ADC) were compared between CNS WHO molecular grade 4 diffuse gliomas (Mol-4 group) and CNS WHO molecular grade 2 or 3 diffuse gliomas (Mol-2/3 group) using Mann-Whitney U tests. Sex, IDH mutation status, and the presence of a gyriform infiltration were compared using Fisher’s exact tests between the 2 groups.

Stepwise logistic regression analysis with Akaike’s information criterion [23] incorporating the ADC histogram profiles with false discovery rate-corrected *p* values < 0.01 and age at diagnosis was performed to identify independent variables for predicting the Mol-4 group. An area under the receiver operating characteristic curve (AUC) derived from the combined model was calculated and used to determine the optimal cutoff values to distinguish the two groups based on the highest Youden index (sensitivity + specificity -1). In addition, a leave-one-out cross-validation (LOOCV) strategy using the identified variables was employed to assess the precision, recall, and F1 score [24]. The lower and upper 95% confidence intervals (95%CI) were calculated using a 1000-time bootstrap sampling [25]. We performed the same analyses for the groups without oligodendrogliomas (i.e., the Mol-4 astrocytomas vs. Mol-2/3 astrocytomas). Two-sided *p* values < 0.05 were considered statistically significant. All statistical analyses were performed using R (version 4.1.1; R Foundation for Statistical Computing) or Matlab (release 2018a, MathWorks).

## Results

### Mol-4 and Mol-2/3 glioma groups

The demographic and pathological findings of the Mol-4 and Mol-2/3 groups are summarized in Table 2. Among the 63 patients, 18 patients (median age, 64.5 [range, 37–84] years; 8 men) were classified as the Mol-4 group, and 45 patients (median age, 40 [range, 18–73] years; 23 men) were classified as the Mol-2/3 group (Table 2). Patient age was significantly higher in the Mol-4 group than in the Mol-2/3 group (*p* < 0.001). One and 42 patients had IDH-mutant gliomas in the Mol-4 and Mol-2/3 groups, respectively (Mol-4 group, 1/18 [5.6%] vs. Mol-2/3 group, 42/45 [93.3%], *p* < 0.0001). *CDKN2A/B* homozygous deletion was found in one patient (2.3%) with IDH-mutant glioma. A gyriform infiltration was detected in two patients (11.1%) in the Mol-4 group and one patient (2.2%) in the Mol-2/3 group (*p* = 0.26).

**Table 2:** Demographic and pathological findings

	Total	Mol-4 group	Mol-2/3 group	FDR-corrected *p*-value
Number	63	18	45	
Age (year; median, range)	45 (18–84)	64.5 (37–84)	40 (18–73)	< 0.001*
Sex (male:female)	31 : 32	8 : 10	23 : 22	> 0.99
Period between MRI and biopsy/surgery (day; median, range)	12 (0–74)	12 (0–74)	14 (0–67)	> 0.99
IDH-mutant:IDH-wildtype	43 : 20	1 : 17	42 : 3	< 0.001*
1p19q co-deleted (among IDH-mutant)	18/43 (41.9%)	0/1	18/42 (42.9%)	
*CDKN2A/B* homozygous deletion (among IDH-mutant)	1/43 (2.3%)	1/1 (100%)	0/42	
+7/−10 (among IDH-wildtype)	9/15 (60.0%)	9/13 (69.2%)	0/2	
*EGFR* amplification (among IDH-wildtype)	6/11 (54.5%)	6/9 (66.7%)	0/2	
*TERT* promoter mutation (among IDH-wildtype)	12/17 (70.6%)	12/14 (85.7%)	0/3	

*FDR*, false discovery rate; *IDH*, isocitrate dehydrogenase; *EGFR*, epidermal growth factor receptor; *TERT*, telomerase reverse transcriptase; *CNS*, central neural system; *WHO*, World Health Organization

^*^Statistically significant

### ADC histogram analysis

The 90th percentile, median, mean, 10th percentile, and entropy of ADC were significantly lower in the Mol-4 group than in the Mol-2/3 group (Table 3). Kurtosis and skewness of ADC were significantly higher in the Mol-4 group than in the Mol-2/3 group.

**Table 3:** ADC histogram profiles

Parameters (median, interquartile range)	Mol-4 group (*n* = 18)	Mol-2/3 group (*n* = 45)	FDR-corrected *p*-value
nADCmax	3.88 (3.32–4.15)	4.03 (3.57–4.43)	0.59
nADC90perc	1.66 (1.53–1.97)	2.14 (1.98–2.57)	< 0.001*
nADCmedian	1.29 (1.25–1.50)	1.68 (1.51–1.86)	< 0.001*
nADCmean	1.34 (1.27–1.54)	1.70 (1.55–1.87)	< 0.001*
nADC10perc	1.08 (1.02–1.14)	1.15 (1.12–1.22)	0.014*
nADCmin	0.36 (0.11–0.65)	0.46 (0.19–0.67)	> 0.99
nADCkurtosis	8.17 (5.84–9.65)	3.22 (2.50–5.88)	< 0.001*
nADCskewness	1.42 (0.88–2.03)	0.34 (0.10–1.13)	0.046*
nADCentropy	6.17 (5.86–6.49)	6.77 (6.45–6.97)	< 0.001*

*ADC*, apparent diffusion coefficient; *FDR*, false recovery rate; nADCmax/90perc/median/mean/10perc/min/kurtosis/skewness/entropy, maximum/90 percentile/median/mean/10 percentile/minimum/kurtosis/skewness/entropy of normalized apparent diffusion coefficient

^*^Statistically significant

### Multivariate analysis

**Fig. 1:**
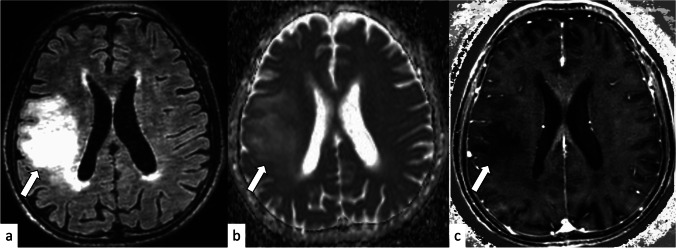
An 84-year-old man with IDH-wildtype histologically lower-grade astrocytic glioma with molecular evidence of CNS WHO grade 4 (concurrent gain of the whole chromosome 7 and loss of the whole chromosome 10 and *TERT* promoter mutation). The tumor shows hyperintensity on the fluid-attenuated inversion recovery image (**a**), and the median and entropy of the normalized apparent diffusion coefficient are 1.28 and 6.19, respectively (**b**). No evidence of necrosis or contrast enhancement is found on the subtraction T1-weighted image (**c**)

**Fig. 2:**
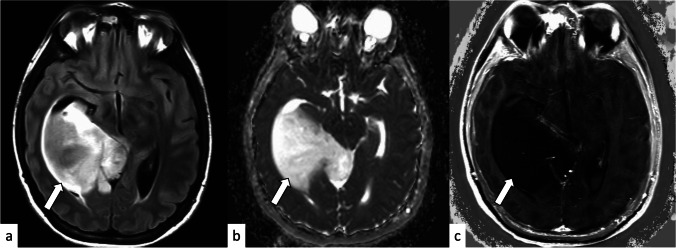
A 26-year-old woman with IDH-mutant histologically lower-grade astrocytoma without homozygous deletions of *CDKN2A/B*. The tumor shows hyperintensity on the fluid-attenuated inversion recovery image (**a**), and the median and entropy of the normalized apparent diffusion coefficient are 2.37 and 6.89, respectively (**b**). No evidence of necrosis or contrast enhancement is found on the subtraction T1-weighted image (**c**)

The age at diagnosis and entropy of ADC were selected for the stepwise multivariate logistic regression analysis, and both of the parameters were identified as independent predictors of the Mol-4 group with AUC of 0.92 (Table 4). The mean precision, recall, and F1 score using LOOCV and 1000-bootstrapping (mean (95%CI)) were 0.79 (0.78–0.79), 0.72 (0.71–0.72), and 0.74 (0.74–0.75), respectively. The representative images of the tumors in the Mol-4 group and the Mol-2/3 group are shown in Fig. 1 and Fig. 2, respectively.

**Table 4:** Multivariate logistic regression analysis

	Multivariate			
Parameter	Odds ratio (lower 95%CI, upper 95%CI)	*p*-value	Standardized regression coefficient	AUC (lower 95%CI, upper 95%CI)
Age	1.11 (1.04–1.18)	0.0011*	1.71 ± 0.52	
nADCentropy	0.090 (0.013–0.64)	0.016*	−1.12 ± 0.47	
(Age + nADCentropy) model				0.92 (0.84–0.995)

*CI*, confidence interval; *FDR*, false discovery rate; *AUC*, area under the receiver operator characteristic curve; *nADCentropy*, normalized entropy of apparent diffusion coefficient

^*^Statistically significant

### Subclass analyses between the Mol-4 and Mol-2/3 astrocytomas

The demographic and pathological findings and the results of ADC histogram analyses are summarized in Supplementary Tables 1 and 2, respectively. Age at diagnosis was significantly higher, and the rate of IDH-mutant tumors was significantly lower in the Mol-4 astrocytomas compared with the Mol-2/3 counterparts (median 64.5 [range, 37–84] years vs. 33 [18–60] years, *p* < 0.001; IDH-mutant vs. IDH-wildtype: 1:17 vs. 24:3, *p* < 0.001). The age at diagnosis and median of ADC were selected for the stepwise multivariate logistic regression analysis, and the AUC of the model was 0.98 (0.94–1) (Supplementary Table 3). The mean precision, recall, and F1 score using LOOCV and 1000-bootstrapping (mean (95%CI)) were 0.86 (0.86–0.87), 0.85 (0.85–0.86), and 0.86 (0.85–0.86), respectively. Although the median of ADC tended to be lower in the Mol-4 astrocytomas, the *p*-value did not reach statistical significance.

## Discussion

In this study, we compared ADC histogram profiles in histologically lower-grade adult diffuse gliomas between the presence (the Mol-4 group) and absence (the Mol-2/3 group) of molecular evidence for CNS WHO grade 4 in accordance with WHO CNS5. Many ADC histogram profiles were significantly different between the two groups, and the entropy of normalized ADC and age at diagnoses were found as independent predictive factors for the Mol-4 group with an AUC of 0.92. On the other hand, while several ADC histogram profiles were significantly different between the groups, only age at diagnosis was the independent predictive factor for the Mol-4 astrocytoma group in the comparison between the Mol-4 and Mol-2/3 astrocytomas.

In the present study, we found that 1st-order ADC histogram profiles (i.e., maximum, 90th percentile, median, mean, and 10th percentile) were significantly lower in the Mol-4 group than in the Mol-2/3 group. These results were similar to the results of the subclass analyses of astrocytomas. Traditionally, a lower ADC has been considered to be associated with increased cellularity [26]. Furthermore, many studies have reported that lower ADC is a negative prognostic biomarker in gliomas independent of the grade. In the meta-analysis of studies published between 2006 and 2010, Zulfigar et al. [27] demonstrated that a low minimum ADC was associated with a shorter survival rate in patients with grades III and IV diffuse glioma when cases were stratified according to the grades. Cuccarini et al. [28] reported that a low minimum ADC was associated with a shorter overall survival in patients with grades II and III diffuse glioma when cases were stratified according to the grades. Hilario et al. [29] found that older age and a low median ADC were associated with shorter overall survival independent of tumor grade in their study including patients with grades II, III, and IV diffuse gliomas. Despite the consistency of the results, it should be noted that diffuse gliomas in these studies were diagnosed according to the previous WHO classifications without the latest molecular updates; thus, histologically lower-grade molecular grade 4 diffuse gliomas (i.e., the Mol-4 group) were likely included in their grades II or III diffuse glioma groups [26–29]. Therefore, the variations in ADC and prognosis among the “same” grade diffuse gliomas in these studies may have been affected, at least in part, by the differences in the presence or absence of the molecular features of grade 4, as shown in the present study.

The multivariate stepwise logistic regression analysis revealed that age at diagnosis and the entropy of normalized ADC were independent parameters for predicting the Mol-4 group. The second-order parameters derived from the whole tumor ADC histograms, namely kurtosis, skewness, and entropy, have been evaluated for tumor grading and for estimating the proliferative potential of the tumors [30–32]. Kurtosis represents the distribution peakedness of the intensity for the parameter within the tissue. Skewness represents the distribution asymmetry. Entropy represents the intensity predictability of the parameter and reflects textural variation. Differences in the entropy of normalized ADC in the present study may have reflected the difference in microstructural heterogeneity between the two groups. Although the *p*-value of the entropy of ADC did not reach statistical significance and only age at diagnosis was an independent predictive factor for the Mol-4 astrocytoma group in the subclass analysis without oligodendrogliomas, the entropy of ADC was also selected in the stepwise selection. The higher patient age in the Mol-4 group compared with the Mol-2/3 group was in line with the study by Reuss et al. [33], although they did not compare the patient age statistically. Further studies with a larger number of cases are warranted to verify these results.

While ADC histogram profiles were shown to be promising imaging biomarkers in differentiating Mol-4 group from Mol-2/3 group, we could not verify the difference in the frequency of the gyriform infiltration on FLAIR imaging between the two groups. The gyriform infiltration was reported to be present in 16/31 patients (51.6%) in the Mol-4 group and 0/151 patients (0%) in the Mol-2/3 group with a statistically significant difference in the frequency by a French group [18]. The discrepancy between the results of their study and the present study should be further investigated by another group since other than one study by the same French group with the patients from the same institution [34] and the present study, no study has examined the frequency of the gyriform infiltration in the Mol-4 group. It should be noted that although all of their Mol-4 group tumors corresponded to lower-grade IDH-wildtype astrocytomas in the previous WHO 2016 classification [18], the high frequency of gyriform infiltration in lower-grade IDH-wildtype astrocytomas has not been reported from other groups.

We recognize several limitations in the present study. First, this was a single institutional retrospective study, which limited the number of patients. Second, MRI examinations were performed using multiple scanners at different field strengths. However, to minimize the influence of the machine differences, we normalized ADC parameters. Further, ADC is theoretically insensitive to the difference in field strength, even though a higher field strength may benefit from a higher contrast-to-noise ratio [35]. Third, most of the Mol-4 group consisted of IDH-wildtype diffuse gliomas in the present study. However, this reflects the real-world frequency because we included consecutive patients, and the similar frequency of IDH-wildtype diffuse gliomas has been reported in other studies [18, 33]. Finally, we could not evaluate the correlation between histogram profiles and patient survival. The association with prognosis needs to be investigated in future studies.

## Conclusion

Whole tumor histogram-derived ADC profiles may be promising imaging biomarkers on preoperative brain MRI with significant differences between the presence and absence of molecular features for grade 4 in histologically lower-grade adult diffuse gliomas.

### Supplementary Information

Below is the link to the electronic supplementary material.

Supplementary file1 (PDF 161 KB)

## Abbreviations

+7/-10: Concurrent gain of the whole chromosome 7 and loss of the whole chromosome 10
AUC: Area under the receiver operating characteristic curve
EGFR: Epidermal growth factor receptor
FDR: False discovery rate
IDH: Isocitrate dehydrogenase
TERT: Telomerase reverse transcriptase

## Declarations

### Guarantor

The scientific guarantor of this publication is Prof. Toshio Moritani.

### Statistics and biometry

No complex statistical methods were necessary for this paper.

### Study subjects or cohorts overlap

No study subjects or cohorts have been previously reported.

### Methodology

• retrospective

• case-control study

• performed at one institution
