# Supplementary material for: Diffusion histogram profiles predict molecular features of grade 4 in histologically lower-grade adult diffuse gliomas following WHO classification 2021
Source: Eur Radiol. 2023 Aug 15;34(2):1367–75. doi: 10.1007/s00330-023-10071-x (PMC10853353; doi:10.1007/s00330-023-10071-x)
Supplement: Supplementary file 1 — Supplementary file1 (PDF 161 KB) [file 330_2023_10071_MOESM1_ESM.pdf]

Diffusion histogram profiles predict molecular features of grade 4 in histologically lower-grade adult diffuse gliomas following WHO classification 2021

ELECTRONIC SUPPLEMENTARY MATERIAL

Supplemental Table 1. Demographic and pathological findings of astrocytomas

	Total	Mol-4 astrocytoma	Mol-2/3 astrocytoma	FDR-corrected P-value
Number	45	18	27	
Age (year; median, range)	44 (18–84)	64.5 (37–84)	33 (18–60)	<0.001*
Sex (Male : Female)	23 : 22	8 : 10	15 : 12	>0.99
Period between MRI and biopsy/surgery (day; median, range)	12 (0–74)	12 (0–74)	14 (0–67)	>0.99
IDH-mutant: IDH-wildtype	25 : 20	1 : 17	24 : 3	<0.001*
CDKN2A/B homogyzous deletion (IDH-mutant)	1/25 (4.0%)	1/1 (100%)	0/24	
+7/-10 (IDH-wildtype)	9/15 (60.0%)	9/13 (69.2%)	0/2	
EGFR amplification (IDH-wildtype)	6/11 (54.5%)	6/9 (66.7%)	0/2	
TERT promoter mutation (IDH-wildtype)	12/15 (60.0%)	12/14 (86.7%)	0/3	

FDR, false discovery rate; IDH, isocitrate dehydrogenase; EGFR, epidermal growth factor receptor; TERT, telomerase reverse transcriptase; CNS, central neural system; WHO, world health organization
*Statistically significant

Supplementary Table 2. ADC histogram profiles of astrocytomas

Parameters (median, interquartile range)	Mol-4 astrocytoma (n = 18)	Mol-2/3 astrocytoma (n = 27)	FDR-corrected P-value
nADCmax	3.88 (3.32–4.15)	4.05 (3.77–4.32)	0.30
nADC90perc	1.66 (1.53–1.97)	2.43 (2.02–2.65)	<0.001*
nADCmedian	1.29 (1.25–1.50)	1.77 (1.57–1.95)	<0.001*
nADCmean	1.34 (1.27–1.54)	1.81 (1.59–1.90)	<0.001*
nADC10perc	1.08 (1.02–1.14)	1.15 (1.13–1.19)	0.014*
nADCmin	0.36 (0.11–0.65)	0.33 (<0.1–0.60)	>0.99
nADCkurtosis	8.17 (5.84–9.65)	2.58 (2.15–4.39)	<0.001*
nADCskewness	1.42 (0.88–2.03)	0.26 (0.10–0.69)	0.020
nADCentropy	6.17 (5.86–6.49)	6.77 (6.39–7.05)	<0.001*

ADC, apparent diffusion coefficient; FDR, false recovery rate; nADCmax/90perc/median/mean/10perc/min/kurtosis/skewness/entropy/low/high, normalized maximum/90 percentile/median/mean/10 percentile/minimum/ kurtosis/skewness/entropy/low/high of apparent diffusion coefficient

*Statistically significant

Supplementary Table 3. Multivariate logistic regression analysis of astrocytomas

Parameter	Multivariate			AUC (lower 95%CI, upper 95%CI)
	Odds ratio (lower 95%CI, upper 95%CI)	P-value	Standardized regression coefficient	
Age	1.19 (1.06–1.33)	0.0027*	3.04 ± 1.01	
nADCmedian	0.023 (0.00022–2.44)	0.11	-1.42 ± 0.89	
(Age + nADCmedian) model				0.98 (0.94–1)

CI, confidence interval; FDR, false discovery rate; AUC, area under the receiver operator characteristic curve

nADCentropy, normalized entropy of apparent diffusion coefficient

*Statistically significant
